# Co-doped g-C_3_N_4_ nanotube decorated separators mediate polysulfide redox for high performance lithium sulfur batteries†

**DOI:** 10.1039/d2na00645f

**Published:** 2022-11-22

**Authors:** Zunhao Fan, Mengting Zhu, Shungui Deng, Yanhua Chen, Yue Zhao, Mengyuan Qin, Guiyuan Ma, Jinghua Wu, Xing Xin

**Affiliations:** a School of Material Science and Chemical Engineering, Ningbo University Ningbo 315211 P. R. China xinxing@nbu.edu.cn; b Ningbo Institute of Materials Technology and Engineering, Chinese Academy of Sciences Ningbo 315201 P. R. China wujh@nimte.ac.cn; c Zhejiang Fashion Institute of Technology Ningbo 315211 Zhejiang P. R. China; d Center of Materials Science and Optoelectronics Engineering, University of Chinese Academy of Sciences Beijing 100049 P. R. China; e Key Laboratory of Material Physics, Ministry of Education, School of Physics and Microelectronics, Zhengzhou University Zhengzhou 450001 China

## Abstract

The main issue with lithium–sulfur (Li–S) batteries is the serious irreversible capacity loss caused by the polysulfide shuttle process. In this work, we propose an electro-catalytic strategy for absorbing and transferring long-chain polysulfides during the redox process, which is the key to improving the utilization of S. Reported here is a Co doped tubular g-C_3_N_4_ (CN) modified separator (Co-TCN@PP), which successfully inhibited the polysulfide shuttle by physical absorption and catalysis, thus facilitating the high utilization of S. Co-TCN with a tube-like structure ensures the uniform dispersion of Co nanoparticles, which provides abundant active sites to absorb polysulfides. Furthermore, Co-TCN exhibits fast reaction kinetics for polysulfide conversion. A Li–S battery with Co-TCN@PP achieves superior rate capacities and a long cycle life (400 times) with capacity fading as low as 0.07% per cycle at a high Li^+^ insertion/extraction rate of 2C. Moreover, electrodes with a high sulfur loading of 5.6 mg cm^−2^ can be realized by adopting the Co-TCN@PP separator.

## Abstract 2

A Co-doped g-C3N4 modified separator captures and converts polysulfides to realize the high utilization of S for Li–S batteries.

## Graphical abstract

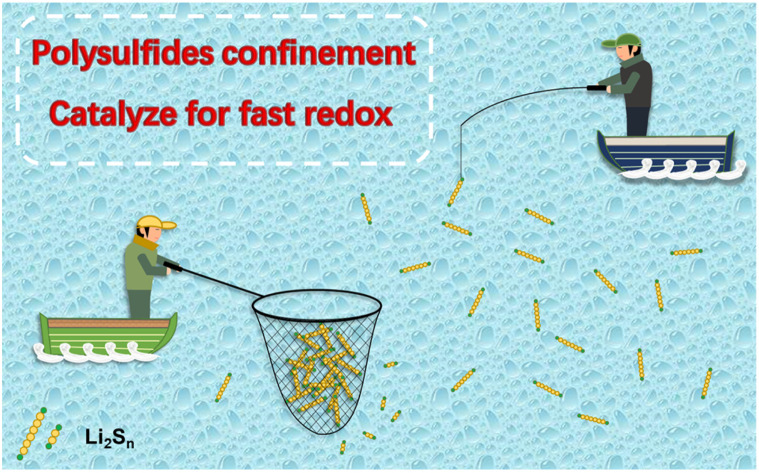

## 1. Introduction

As the capacity of commercialized Li-ion batteries has been approaching the theoretical limit, a new energy system based on lithium–sulfur (Li–S) chemistry is projected to be one of the research hotspots in the energy conversion field due to its highly specific capacity and gravimetric energy density (1675 mA h g^−1^ and 2600 W h kg^−1^).^1–4^ However, the implementation of Li–S batteries has been limited by the poor intrinsic electronic properties of S and the “shuttle effect” of dissolved polysulfides, which challenges the long-term cycling stability and high capacity required for commercial applications.^5–9^ In the past few decades, various host materials like carbon materials, metal oxides, and metal sulfides have been explored as cathodes to confine the sulfur species.^10–15^ These composites worked as excellent electron conducting substrates to enhance the electronic conductivity of almost insulating S, thus improving the capacity performance of Li–S batteries. Nevertheless, these composites are difficult to prevent the polysulfide shuttle effect because the confinement of polysulfides by these composites is a limited physical adsorption process.

Recently, another promising strategy has been directed toward fabricating a functional interlayer or modifying the separator on the cathodic side, which not only prevents the penetration of polysulfides but also expedites the conversion of polysulfides.^16–25^ Carbon materials were first utilized to modify the separator, which can physically confine polysulfides, and at the same time, work as a conducting matrix to catalyze the conversion of polysulfides.^26–28^ However, carbon materials have low affinity to polysulfides, which is unfavorable for trapping polysulfides and thus the shuttle effect may not be well addressed. Recently, Zhang *et al.*^29^ have put forward a strong dipolar–dipolar interaction between polysulfides and the cathode material derived from an electron-rich donor (*e.g.*, pyridine nitrogen), which is favorable to realize a high adsorption. Therefore, N doping of carbon materials is believed to be effective in adsorbing polysulfides. Graphitic carbon nitride (g-C_3_N_4_) possesses a similar architecture to graphite, consisting of continuous tri-*s*-triazine units and amino groups in each layer. Due to its highest nitrogen doping degree and unique band gap which can provide sufficient active sites, g-C_3_N_4_ (CN) is considered as a suitable polysulfide adsorbent material.^30,31^ Density functional theory simulation and first-principles calculation have also demonstrated the electrostatic-induced interaction of CN to polysulfides and the redox kinetic enhancement with distortion of the molecular configuration of polysulfides.^32^ Nevertheless, pristine CN still suffers from a limited surface area due to the stacked layered structure, which is unfavorable to expose sufficient active sites. Moreover, pristine CN delivers poor conductivity, which is unfavorable for catalyzing the S redox reactions. Metal doping is widely used as a necessary method to alter the electronic structure of semiconductors for improving its conductivity.^33–36^ In addition, inspired by conventional catalytic reactions, the electrocatalytic effect to accelerate the conversion reaction of polysulfides has also been introduced into Li–S batteries, which is the key to alleviating the shuttle effect.^37^ Metal compounds with catalytic ability such as Co, Ni, Pt, Fe–Co alloy and W_*x*_C have been explored to eliminate polysulfide shuttle effects.^38–41^

Herein, we successfully synthesized Co doped melamine derived g-C_3_N_4_ with a tube-like structure (Co-TCN) to modify the PP separator for preventing polysulfide shuttle. Co-TCN provides a high surface area and conductivity, which not only facilitate the exposure of the active sites to adsorb polysulfides but also improve the catalysis of polysulfide conversion. Therefore, the Co-TCN modified separator (Co-TCN@PP) could effectively hinder the shuttling effect of polysulfides and improve the electrochemical properties of Li–S batteries. As a result, electrodes with a high S loading of 5.6 mg cm^−2^ can be realized. Moreover, a Li–S battery with the Co-TCN@PP separator exhibits excellent rate capacities of 1304.1, 1174.4, 1021.7, 942.7 and 863.6 mA h g^−1^ at 0.1, 0.2, 0.5, 1 and 2C, respectively, which is higher than that of pristine CN. At a high current density of 2C, the Li–S battery with the Co-TCN@PP separator maintains 659.7 mA h g^−1^ after 400 cycles, corresponding to a low capacity fading of 0.07% per cycle.

## 2. Results and discussion

**Fig. 1:**
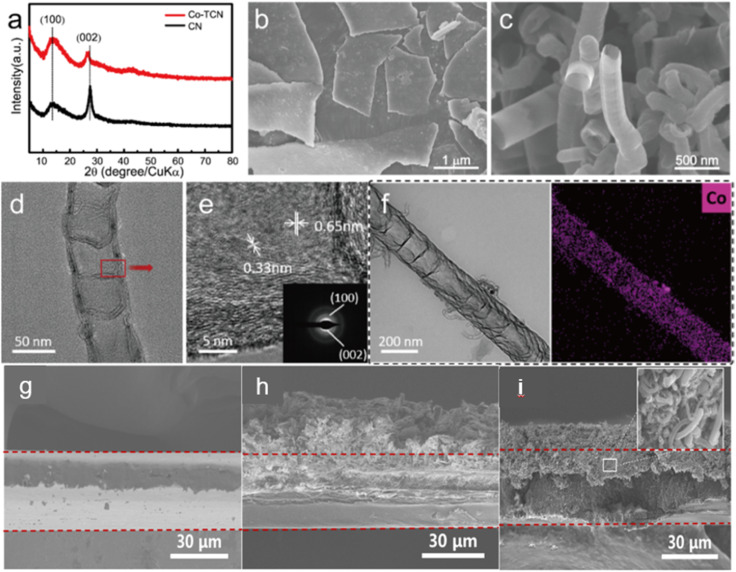
(a) XRD of CN and Co-TCN. SEM of (b) CN and (c) Co-TCN. (d) TEM, (e) HTEM and (f) STEM-EDX mapping of Co-TCN. The cross-section SEM images of (g) PP, (h) CN@PP and (I) Co-TCN@PP.

The XRD patterns of both CN and Co-TCN are presented in Fig. 1a. The characteristic peaks at 13.2° and 27.5° are typically assigned to the interlayer stacking peak (002) and in-plane peak (100) of CN, respectively. Besides, the lower intensity and the slight shifting of the (002) peak of Co-TCN demonstrate the disorder of the graphitic structure with low crystallinity, which can be attributed to the tubular structure and the Co doping.^42–46^ No obvious peaks assigned to the Co crystals can be found in the Co-TCN sample, which indicates the poor crystallization of Co. Fig. 1b and c compare the morphology of CN and Co-TCN. CN has a typical flaky structure with two dimensional layers as shown in Fig. 1b. These layers stacked together to form sheets with a certain thickness. After Co intaking, the CN flakes curled into a tube-like structure as shown in Fig. 1c. The Co atoms will partly replace the atoms of C_3_N_4_ and thus induce an uneven stress of the layer. The layer therefore turns into a tube-like structure.^47^ The Co-TCN tubes with a hollow interior are about 200 nm in diameter, which is well imaged by TEM as shown in Fig. 1d. The corresponding HTEM as shown in Fig. 1e demonstrates an interplanar crystal spacing of 0.33 nm and 0.65 nm corresponding to the (100) and (002) crystal faces of CN. The elemental mapping in Fig. 1f reveals the uniform distribution of Co in the CN, while no obvious particles can be found. The thickness of CN and Co-TCN on the PP separator was 18.1 and 11.6 μm, respectively (Fig. 1h and i). From Fig. S4a and S4b,† Co-TCN possesses a high BET surface area (206.61 m^2^ g^−1^), which is about 22 times higher than that of CN (9.29 m^2^ g^−1^). The typical V type isotherms and BJH distribution confirm the existence of mesopores in Co-TCN composites. It is quite clear that compared with CN, more active sites were exposed after the Co doping. The appearance of sp^2^ carbon of Co-TCN upon high temperature heating is believed to contribute to the improvement of the electron conductivity. We measured the sheet resistance by the four-point probe method as shown in Fig. S4c.† The electron conductivity of Co-TCN and CN is 0.447 and 0.313 × 10^−8^ S cm^−1^, respectively. The higher conductivity of Co-TCN than that of CN which facilitates the catalysis of S redox reactions can be attributed to the Co doping.

**Fig. 2:**
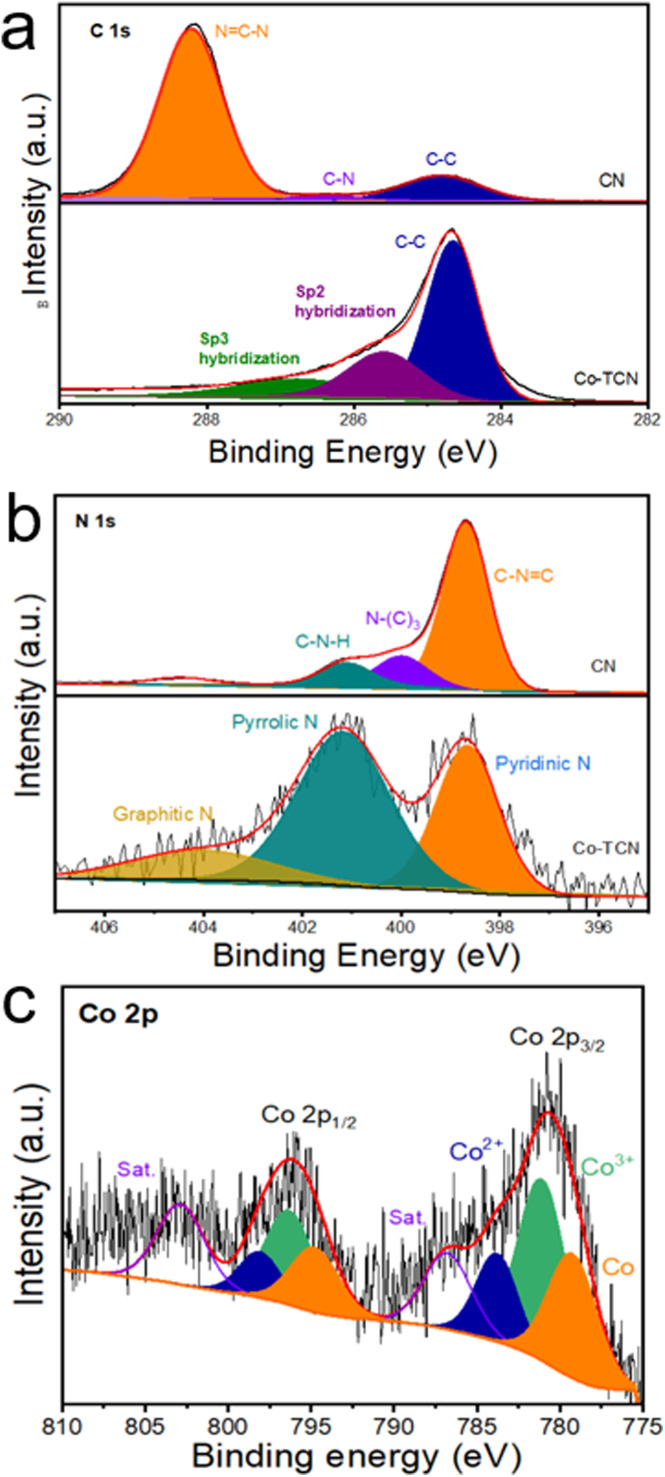
High-resolution XPS spectra of (a) C 1s, (b) N 1s, (c) O 1s and (d) Co 2p for CN and Co-TCN samples.

XPS was carried out to investigate the chemical state of C, N and Co in CN and Co-TCN composites (Fig. 2 and S2†). The XPS survey spectra (Fig. S2†) disclose the existence of Co in Co-TCN, which cannot be detected in XRD. As shown in Fig. 2a, C 1s of CN displays two broad peaks corresponding to N=C–N (288.2 eV) and C–C (284.8 eV). By contrast, the decreases intensity of N=C–N and the increased intensity of C–C in Co-TCN indicate the increased degree of graphitization of carbon. Moreover, after high temperature treatment, the other peaks at 285.6 and 286.7 eV can be identified as predominantly Sp^2^ and Sp^3^ hybridization of C and N.^46^ The N 1s spectrum in Fig. 2b of CN could be divided into three characteristic peaks, attributed to C=N–C (398.7 eV), N–(C)_3_ (399.9 eV) and C–N–H (401.2 eV). After Co doping, two peaks of N are detected, which represent pyridine (398.6 eV) and pyrrole N (401.2 eV).^46^ The Co spectrum in Co-TCN displays two peaks, which represent the spin orbits of Co 2p_3/2_ and Co 2p_1/2_, respectively. In addition to the satellite peaks located at 786.7 and 802.8 eV, the sharp peak at 779.1 eV originates from Co^0^. The peaks at 781.2and 796.3 eV are ascribed to Co^3+^, while the peaks centered at 783.6 and 798.1 eV originated from Co^2+^. The coexistence of Co^2+^ and Co^3+^ indicates the oxidation of the sample.

**Fig. 3:**
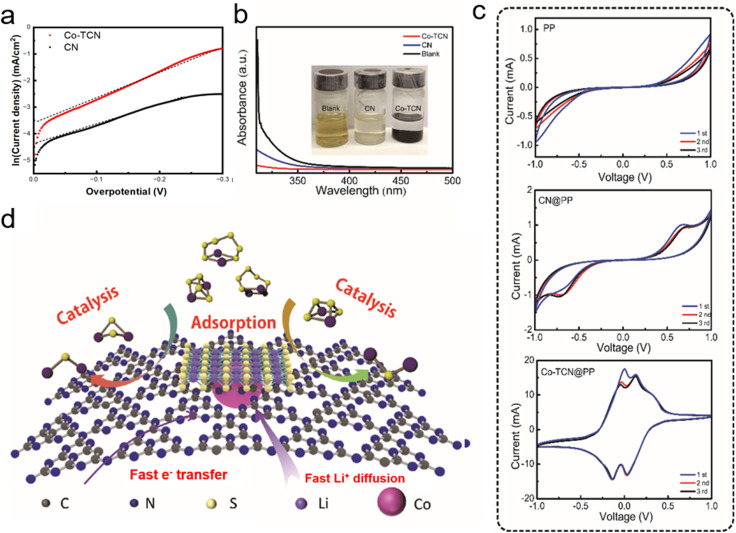
(a) The Tafel plots of the Li_2_S_6_ solution redox based on different host materials tested by LSV. The dotted line was fitted to calculate the exchange current density. (b) UV-vis spectra of Li_2_S_6_ solution before and after adsorption. The inset shows the optical comparison. (c) The CV of the symmetric cells based on different electrodes. (d) The mechanism illustration of the Co-TCN@PP interlayer during the charge–discharge process.

A linear scanning voltammetry (LSV) method was used to elucidate exchange current with Li_2_S_6_ catholyte solution in a three-electrode battery to analyze the difference of dynamic behavior between CN and Co-TCN (Fig. S3†). Here, the single-redox behavior of L_2_S_6_ was designed to represent the polysulfide disproportionation reactions. As obtained from the Tafel plots as shown in Fig. 3a, according to the Butler–Volmer equation (*i* = *i*_0_(e^(1−β)*fη*^ − e^−β*fη*^)), which can be written as the Tafel equation: ln *i* = (1 − β)*fη* + ln *i*_0_, when the overpotential *η* is close to 0, a higher exchange current density *i*_0_ of Co-TCN (0.032 mA cm^−2^) than that of CN (0.0125 mA cm^−2^) can be obtained, which demonstrates the superior conversion kinetics of Co-TCN. Furthermore, the interactions between Li_2_S_6_ and CN and Co-TCN can be illustrated by the color variation as shown in Fig. 3b (inset). There is no color change in the blank sample without any addition after Li_2_S_6_ addition. In contrast, CN and Co-TCN present varying degrees of color loss. It was further proved by ultraviolet-visible (UV-vis) spectra (Fig. 3b) that the Li_2_S_6_ solution has a typical absorbance peak of S^4+^/S^6+^ at around 350 nm, which declines significantly after adsorption by Co-TCN, indicating that the concentration decreases.^48^ From the CV curves of the symmetric cells in a Li_2_S_6_ electrolyte (0.2 M) in DOL/DME as shown in Fig. 3c, a pair of redox peaks at −0.7 V and 0.7 V is visible in CN@PP. In contrast, two redox peaks around −0.2 V and 0.2 V can be found in the Co-TCN@PP cell, demonstrating that Co-TCN has much higher activity and reversibility toward polysulfide conversion (Fig. 3d). The above results show that Co-TCN has a great ability to adsorb and transfer polysulfides. As illustrated in Fig. 3d, Co-TCNs on the separator act as a trap, which can absorb polysulfides and then promote its transformation to S. The shuttle phenomenon was thus terminated on the cathodic side.

**Fig. 4:**
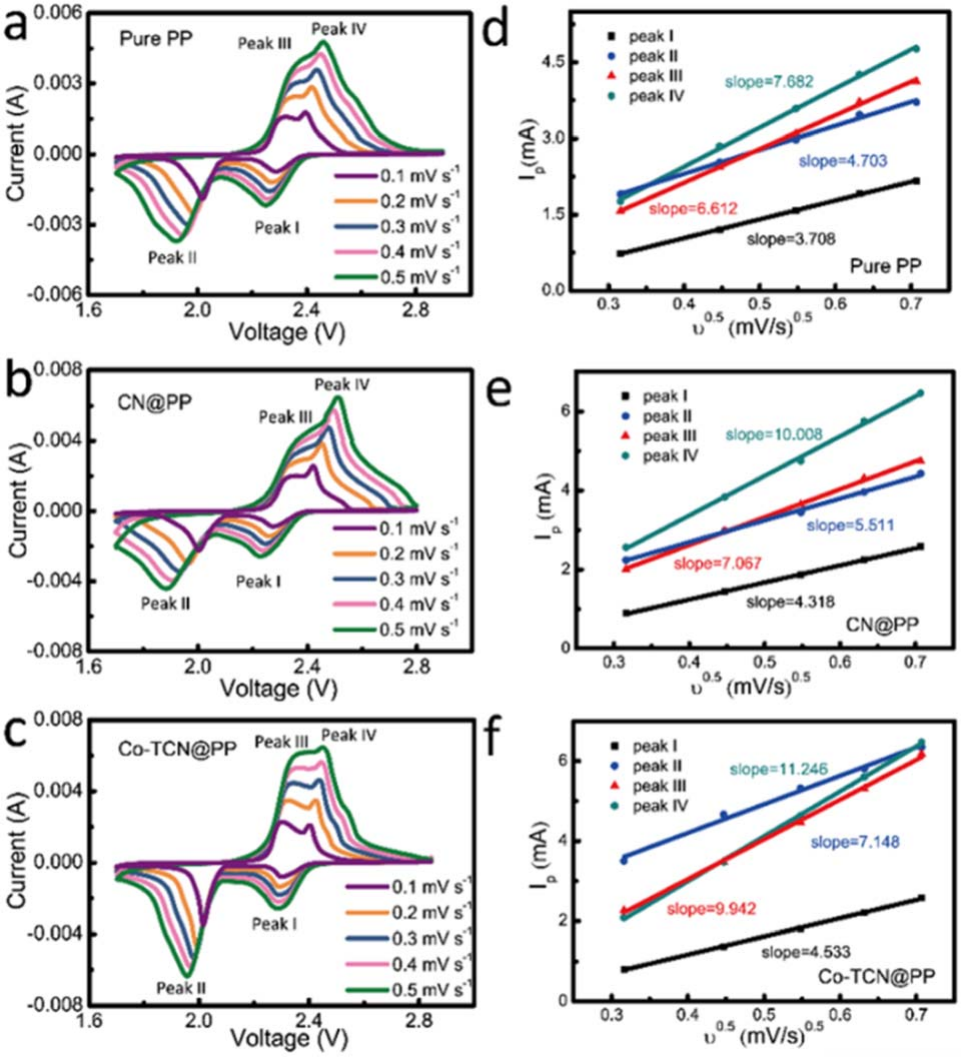
CV curves under different scan rates of (a) PP, (b) CN@PP and (c) Co-TCN@PP. The corresponding linear fits of the peak currents for Li–S batteries with (d) PP, (e) CN@PP and (f) Co-TCN@PP.

In order to validate the electrocatalytic activity of PP, CN@PP and Co-TCN@PP separators, CVs were recorded from 0.1 to 0.5 mV s^−1^ as shown in Fig. 4a–c. Two characteristic reduction peaks around 1.9–2.1 V and 2.2–2.3 V corresponding to the formation of polysulfides and the final product Li_2_S can be found in all three samples at 0.1 mV s^−1^. Compared with PP and CN@PP separators, Co-TCN@PP exhibits higher and sharper peaks in CVs, which evidence fast reaction kinetics. All the samples exhibit two oxidation peaks at 2.3–2.4 V reflecting the oxidation of Li_2_S to sulfur. It should be noted that the two reduction peaks and oxidation peaks of Co-TCN@PP show a positive shift and negative shift compared with the other two separators, which indicate a higher catalytic effect on the oxidation/reduction of S/Li_2_S corresponding to a decreased electrochemical polarization. The Li^+^ diffusion coefficient can respond to the process of polysulfide redox reactions, and the rapid Li^+^ diffusion is favorable to the conversion reaction of polysulfides.^49,50^Fig. 4d–f show the linear fits of the peak currents for Li–S batteries with PP, CN@PP and Co-TCN@PP, which can evaluate the Li^+^ diffusion coefficient. The relationship between the peak current and the square root of scan rate shown in Fig. 4d–f can be described as the Randles–Sevcik equation: *I*_p_ = 2.69 × 10^5^ × *n*^3/2^ × *A* × *D*^1/2^ × *C* × *ν*^1/2^, where *I*_p_ is the peak current (A), *A* is the area of the electrode (1.13 cm^2^), *n* refers to the number of electrons per specific reaction (*n* = 1 for Li^+^), *ν* is the scan rate (mV s^−1^), and *C* is the concentration of Li^+^ in the material (mol L^−1^). Because the *n*, *A* and *C* are constants, the slope of *I*_P_/*ν*^0.5^ can be plotted to determine the Li^+^ diffusion rate (*D*). As shown in Fig. 4d–f, the Co-TCN@PP separator delivered higher slopes of 4.53, 7.15, 9.94, and 11.25 compared with those of CN@PP (4.32, 5.51, 7.07, and 10.01) and pure PP (3.71, 4.70, 6.61, and 7.68). These results prove that Co-TCN@PP has faster diffusion capabilities and better reaction kinetics.

**Fig. 5:**
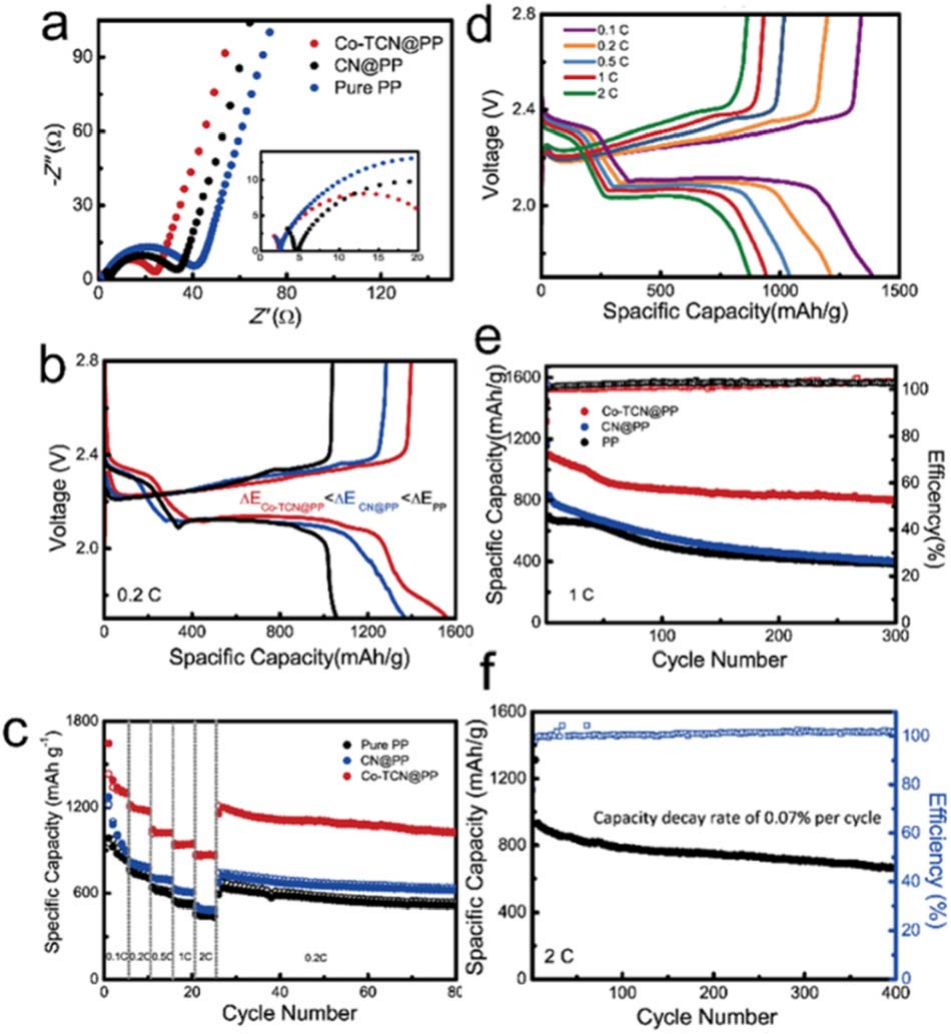
(a) Nyquist plots of Co-TCN@PP, CN@PP and PP. (b) The plateau potential differences and (c) rate performance of PP, CN@PP and Co-TCN@PP-assembled Li–S batteries. (d) The second cycle charge/discharge curves of Co-TCN@PP-assembled Li–S batteries at various rates. Cycling performances of (e) different Li–S batteries at 1.0C and (f) the Co-TCN@PP-assembled Li–S battery at 2C (1C = 1675 mA h g^−1^).

Coin cells were fabricated to compare the electrochemical performances of Li–S batteries with different separators as shown in Fig. 5. The EIS test carried out as shown in Fig. 5a delivered the smallest electrochemical impedance of the Co-TCN@PP based cell, which demonstrates the best charge transfer performance. The discharge/charge curves of the first cycle for all three samples at 0.2C are shown in Fig. 5b. Two distinguish discharge and charge plateaus of all the samples are consistent with CV results, which represent a typical sulfur redox reaction between S_8_ and Li_2_S_2_/Li_2_S. It is noteworthy that the Co-TCN based cell delivered not only the largest initial discharge capacity of 1562 mA h g^−1^ which reflects the highest sulfur utilization but also the smallest gap between charge and discharge plateaus, which indicates the lowest overpotential of 0.156 V.

Fig. 5c shows the rate performances of PP, CN@PP and Co-TCN@PP separator assembled Li–S cells tested from 0.2C to 2C. The Li–S battery with Co-TCN@PP exhibits the highest capacity in each gradient. At a current rate of 0.1C, 1304.1 mA h g^−1^ of the Co-TCN@PP cell can be achieved. When current increased to as high as 2C, a satisfactory capacity of 863.6 mA h g^−1^ can still be obtained. After the high rate test, the Co-TCN@PP cell was retested at a low rate of 0.1C again, and the capacity of Co-TCN@PP cells recovers to 1154.3 mA h g^−1^ and remains at a high level 1025 mA h g^−1^ after 55 cycles. As a comparison, both the CN and pure PP based cells delivered a similar capacity of about 622.5 mA h g^−1^ and 515 mA h g^−1^ after 55 cycles at 0.1C, which is about 40% lower than that of the Co-TCN@PP cell. Fig. 5d presents the galvanostatic charge/discharge curves of the Co-TCN@PP cell after the first active process at different rates. The two distinct discharge and charge plateaus at different current densities demonstrate the stability of the Co-TCN@PP cell. Fig. 5e shows the cycling performances of PP, CN@PP and Co-TCN@PP separator based cells at 1C. Among them, Co-TCN@PP exhibits the most superior performance with an initial capacity of 1116.8 mA h g^−1^. After 300 cycles, the Co-TCN@PP based cell still retains 71.9% of its initial capacity, while CN@PP and PP show 46.7 and 52.9% retention. In addition, even in long-term cycling at 2C (Fig. 5f), Co-TCN@PP can still keep capacity stable over 400 cycles, where the capacity decay rate is only 0.07% for each cycle. Moreover, we demonstrate the feasibility of fabricating electrodes with a high sulfur loading of 5.6 mg cm^−2^. As shown in Fig. S5,† the cathode can sustain a high capacity of above 724.3 mA h g^−1^ over 100 cycles at 1C. To test the application potential, a pouch cell with the Co-TCN@PP separator is assembled.^51^ As shown in Fig. S6,† the pouch cell can still maintain a specific capacity of 547.9 mA h g^−1^ over 250 cycles at 0.2C even when it was bent to 120° and then unfolded after 10 cycles. The galvanostatic discharge–charge curves of the 1st, 11th, 21st and 250th cycles are shown in Fig. S7.† It demonstrated that the folding of the package cell does not degrade the capacity. To further visualize the shuttling inhibition, the dissolution behaviors of polysulfides are determined as shown in Fig. S8.† The Separator is placed between the right Li_2_S_6_ solution and the left blank solvent chambers. For PP and CN@PP separators, the right chamber turned yellow at first and eventually showed the same color as left. However, the right chamber of Co-TCN spparator still kept clear after 24 h. The results verify the blocking ability of Co-TCN for polysulfide diffusion, which benefits from the strong adsorption ability.

**Fig. 6:**
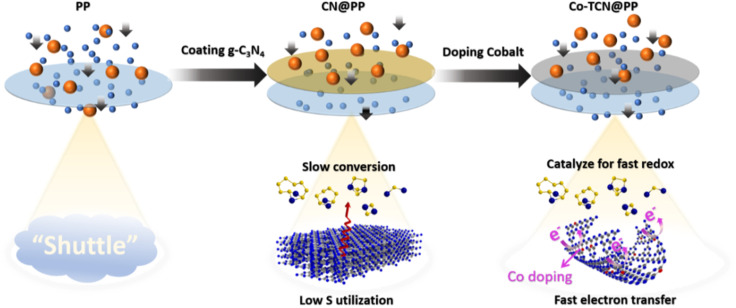
Schematic illustration of the interaction between PP, CN@PP and Co-TCN @PP with polysulfides during the cycling process of the Li–S battery.

The comparison of the effect of Co-TCN @PP for resisting the polysulfide shuttle effect during the cycling process is schematically illustrated in Fig. 6. Compared with PP separator, the CN presented lots of pyridinic N, which is favorable to realize a high adsorption of polysulfides but still impeded by the limited surface area. By contrast, the Co doping into CN changes the morphology from the sheet to a tube-like structure, in which the adsorption sites for catalyzing S redox and transferring electrons can be achieved synchronously, leading to the inhibition of polysulfide shuttle and thus enabling distinct enhancement in the high utilization of S for Li–S batteries.

## 3. Conclusion

In summary, a Co doping CN modified separator for Li–S batteries was rationally designed. The tube-like structure and the Co doping of Co-TCN @PP provide a large surface area and high local conductivity, which hybridizes the advantages of high conductivity and the exposure of active sites, resulting in high physical adsorption and fast reaction kinetics for polysulfide conversion. Therefore, the Li–S battery displays super cycling stability with capacity fading as low as 0.07% per cycle after 400 cycles at 2C and good rate capacities of 1304.1, 1174.4, 1021.7, 942.7 and 863.6 mA h g^−1^ at 0.1, 0.2, 0.5, 1 and 2C, respectively. Moreover, a high sulfur-loading of 5.6 mg cm^−2^ can be realized. This study provides a new strategy to restrict the polysulfide shuttle effect and improve the utilization of sulfur in Li–S batteries.

## 4. Experimental section

### 4.1. Preparation of CN and Co–CN

In a typical synthesis, CN was synthesized by annealing 10 g melamine in a tube furnace at 550 °C for 4 h in an Ar atmosphere with a ramp rate of 5 °C min^−1^. After naturally cooling down to room temperature, the product was then ground and collected and was denoted as CN.

As a comparison, 0.2 g Co(CH3COO)_2_·4H_2_O and 12 g melamine were dissolved in dimethyl sulfoxide (DMSO) solution and shaken vigorously for 24 h. The suspension was then collected after centrifugation and washed with ethanol and deionized water. The sample was then freeze-dried and transferred into a tube furnace for annealing in an Ar atmosphere. The powder was pyrolyzed at 600 °C for 2 h and then carbonized at 900 °C for 1 h. After naturally cooling to room temperature, the powder was then washed in 1 M HCl to remove the isolate Co and dried to obtain the final product, which was denoted as Co-TCN.

### 4.2. Preparation of CN@PP and Co-TCN@PP separators

To prepare the modified separator, the as-prepared Co-TCN and polyvinylidene fluoride (PVDF) were first dispersed in *N*-methyl-2-pyrrolidone (NMP) solvent at a mass ratio of 9 : 1. Then, the mixture was vacuum-filtered to coat onto a PP separator. After being dried at 60 °C in a vacuum, the Co-TCN@PP separator was finally obtained. The mass loading of Co-TCN was controlled at about 1 mg cm^−2^ and the diameter of the separator was 18 mm. The CN@PP separator was prepared in the same way.

### 4.3. Preparation of the sulfur/KB composite electrode

Sublimed sulfur (S) and ketjen black (KB) were homogeneously mixed in a weight ratio of 7 : 3 and annealed at 155 °C for 12 h in an Ar atmosphere. Then, a mixture of the S/KB composite (90%) and PVDF (10%) was dispersed and well-stirred in *N*-methyl pyrrolidone (NMP) solvent. Subsequently, the as-prepared slurry was casted onto aluminum foil. The electrodes were vacuum dried under 60 °C overnight and punched into wafers with a diameter of 13 mm. The sulfur loading of the electrode was controlled at 1 mg cm^−2^. The highest sulfur loading of the electrode can reach 5.6 mg cm^−2^ as shown in Fig. S5.†

### 4.4. Material characterization

The phases and chemical composition of the as-synthesized samples were characterized by X-ray powder diffraction (XRD) on a Bruker D8 advance diffractometer. The surface species and chemical states of CN and Co-TCN were determined by X-ray photoelectron spectroscopy (XPS) measurements (Thermo Scientific K-Alpha) with a monochromatic Al Kα X-ray source (1486.6 eV) and a spot size (400 μm). The XPS data were acquired at room temperature and a lab-based ambient pressure. The XPS spectra were fitted according to the Doniach-Šunjić function. The required parameters are the binding energy, intensity, Lorentzian line width, Gaussian line width, and asymmetry factor.^46,52^ The BET was analyzed by N_2_ adsorption–desorption using an Autosorb-iQ. A Hitachi S4800 field emission scanning electron microscope (SEM) and FEI Tecnai G2 F20 transmission electron microscope (TEM) were used to analyze the microstructure. The sheet resistance was measured by using a four-point probe (CRESBOX).

### 4.5. Battery assembly and electrochemical measurements

The different separators and S/KB as cathodes and Li foil as the anode were assembled into coin cells (CR2032) in an Ar-filled glove box. 1 M lithium bis(trifluoromethanesulfonyl)imide (LiTFSI) in a solvent of 1,2-dimethoxyethane (DME) and 1,3-dioxolane (DOL) (1 : 1 by volume) with the LiNO_3_ additive (1 wt%) was used as the electrolyte. Pouch cells were assembled by using S/KB as the cathode (2 × 2 cm^2^), Co-TCN@PP as the separator (3 × 3 cm^2^) and a Li foil anode (2 × 2 cm^2^, 50 μm of thickness). The S loading for the coin cell and pouch cells was 1 mg cm^−2^. The highest S lading was 5.6 mg cm^−2^. The detailed information on the battery setup is given in Table S1.† Electrochemical impedance spectroscopy (EIS) between 10^6^ and 10 Hz with an amplitude of 15 mV was conducted on a multi-channel potentiostat electrochemical workstation (Solartron 1470E). A multichannel battery test system (LAND CT-2001A, Wuhan Rambo Testing Equipment Co., Ltd) was used to test the galvanostatic discharge–charge and cycling performance of the batteries at different current densities at room temperature within the voltage range of 1.7–2.8 V. The specific capacities were calculated based on the mass of sulfur in the electrode.

### 4.6. Visual observation experiments

Li_2_S_6_ solution was prepared by dissolving Li_2_S and S in the electrolyte in a molar ratio of 1 : 5. The adsorption experiment was conducted by immersing a certain amount of CN and Co-TCN powders in 2 mM Li_2_S_6_ solution. The diffusion experiment was carried out by placing CN@PP and Co-TCN@PP separators between a blank electrolyte and 2 mM Li_2_S_6_ solution.

### 4.7. Linear sweep voltammetry (LSV) test

Co-TCN and PVDF were mixed with a weight ratio of 9 : 1 in NMP solvent to form ink. The uniform ink was coated on carbon paper to prepare the Co-TCN electrode. The LSV test towards Li_2_S_6_ reduction was carried out using Co-TCN or CN as the working electrode and Li foil as both the counter electrode and the reference electrode. The electrolyte contains 0.1 M Li_2_S_6_ in methanol solution. The scan rate of the LSV test was set to 10 mV s^−1^. The electrochemical analysis is based on the Butler–Volmer equation.

### 4.8. Assembly of Li_2_S_6_ symmetric cells and electrochemical measurements

The electrodes were obtained by coating the slurry consisting of KB and PVDF binder (mass ratio: 9 : 1) on Al foil with a mass loading of 1 mg cm^−2^. KB was selected as electrodes for symmetric cells. The symmetric cells were assembled using acetylene black electrodes, PP/CN@PP/Co-TCN@PP separators and a 60 μL electrolyte. The electrolyte included 0.2 M Li_2_S_6_, 1 M LiTFSI and 1 wt% LiNO_3_ in DOL and DME (v:v, 1 : 1). Cyclic voltammetry (CV) was carried out at a scan rate of 5 mV s^−1^ between −1.0 and 1.0 V.

## Conflicts of interest

There are no conflicts to declare.

## Supplementary Material

NA-005-D2NA00645F-s001
