# Supplementary material for: Co-doped g-C3N4 nanotube decorated separators mediate polysulfide redox for high performance lithium sulfur batteries
Source: Nanoscale Adv. 2022 Nov 22;5(2):471–8. doi: 10.1039/d2na00645f (PMC9846446; doi:10.1039/d2na00645f)
Supplement: NA-005-D2NA00645F-s001 [file NA-005-D2NA00645F-s001.pdf]

Electronic Supplementary Material (ESI) for Nanoscale Advances.
This journal is © The Royal Society of Chemistry 2022

Supporting information for

Co-doped g-C_3N_4 nanotubes decorated separators mediate polysulfide redox for high performance lithium sulfur batteries

Zunhao Fan,[a] Mengting Zhu,[a, b] Shungui Deng,[b] Yanhua Chen,[c] Yue Zhao,[b,e] Mengyuan Qin,[a] Guiyuan Ma,[a] Jinghua Wu,[b, d] * Xing Xin[a*]

a School of Material Science and Chemical Engineering, Ningbo University, Ningbo 315211, P. R. China

b Ningbo Institute of Materials Technology and Engineering, Chinese Academy of Sciences, Ningbo 315201, P. R. China

c Zhejiang Fashion Institute of Technology, Ningbo 315211, Zhejiang, P. R. China

d Center of Materials Science and Optoelectronics Engineering, University of Chinese Academy of Sciences, Beijing 100049, P. R. China

e Key Laboratory of Material Physics, Ministry of Education, School of Physics and Microelectronics, Zhengzhou University, Zhengzhou 450001, China

*Corresponding authors: xinxing@nbu.edu.cn, wujh@nimte.ac.cn

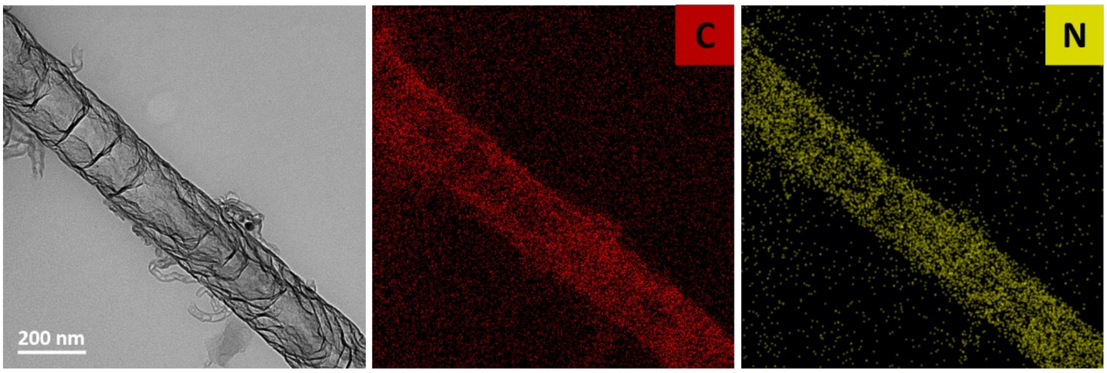

Fig. S1. STEM-EDX mapping of Co-TCN

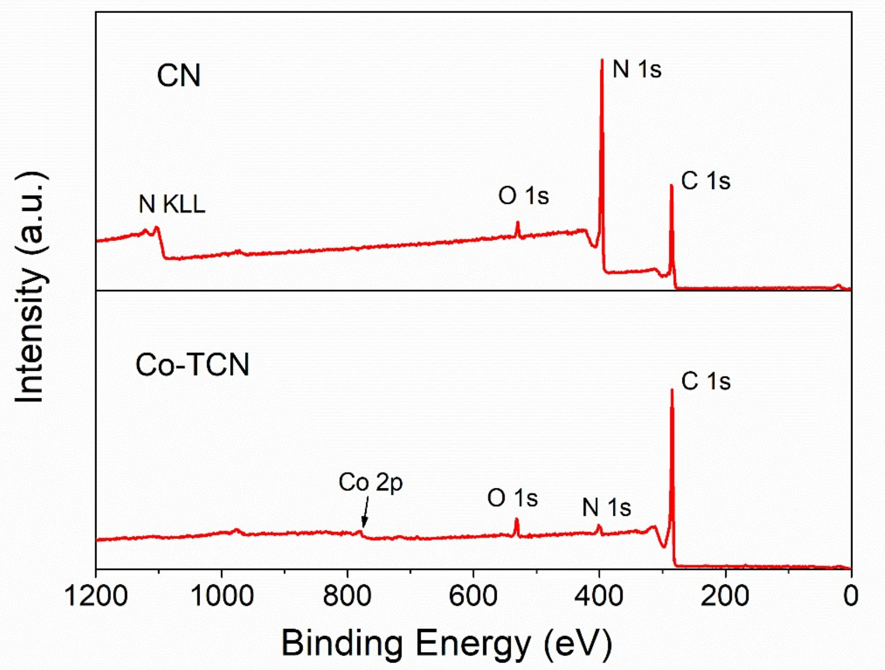

Fig. S2. XPS spectra of full spectrum for Co-TCN sample.

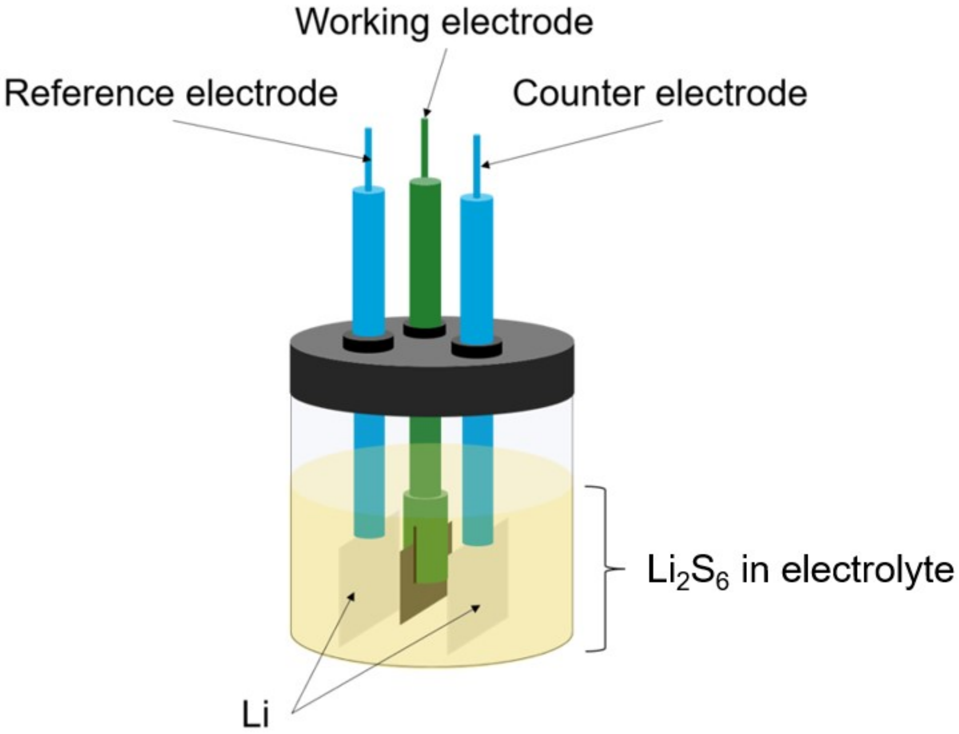

Fig. S3. Diagram of elucidated by exchange current measurements using linear scanning voltammetry (LSV) with a Li_2S_6 solution in a three-electrode cell.

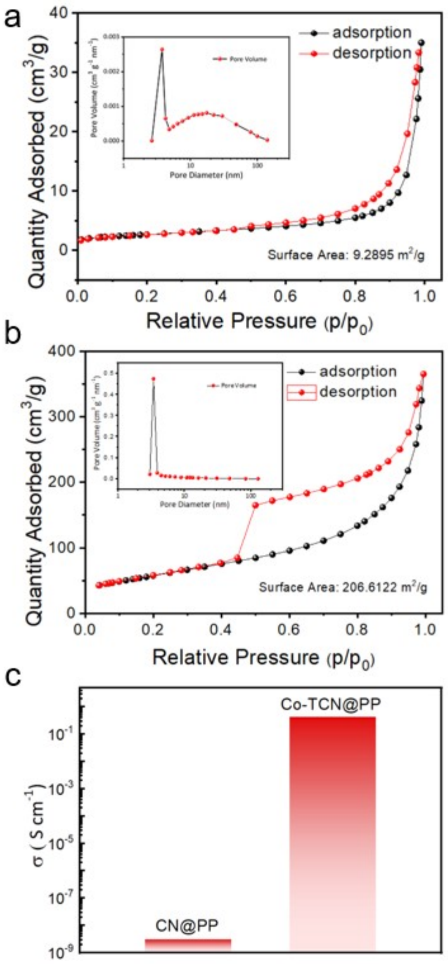

Fig. S4. N_2 adsorption/desorption isotherms of g) CN and h) Co-TCN. i) Conductivity of CN and Co-TCN.

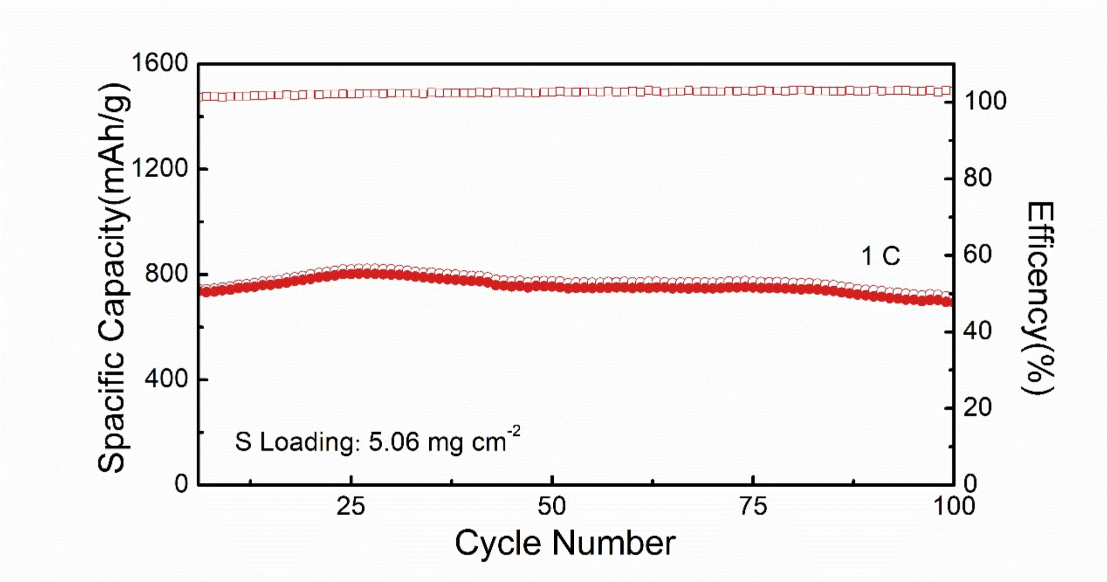

Fig. S5. Cycling performances of Co-TCN with high loading at current density of 1 C.

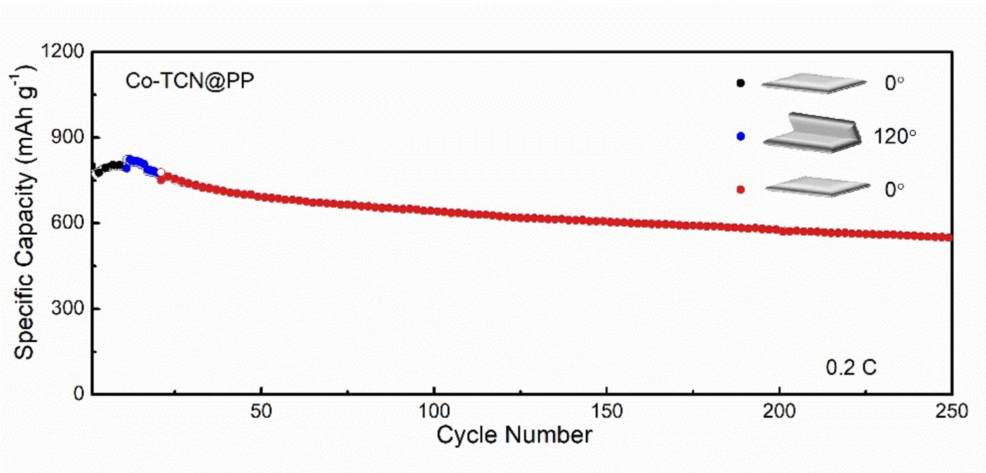

Fig. S6. Cycling performances of Co-TCN assembled Li-S pouch cell at 0.2 C.

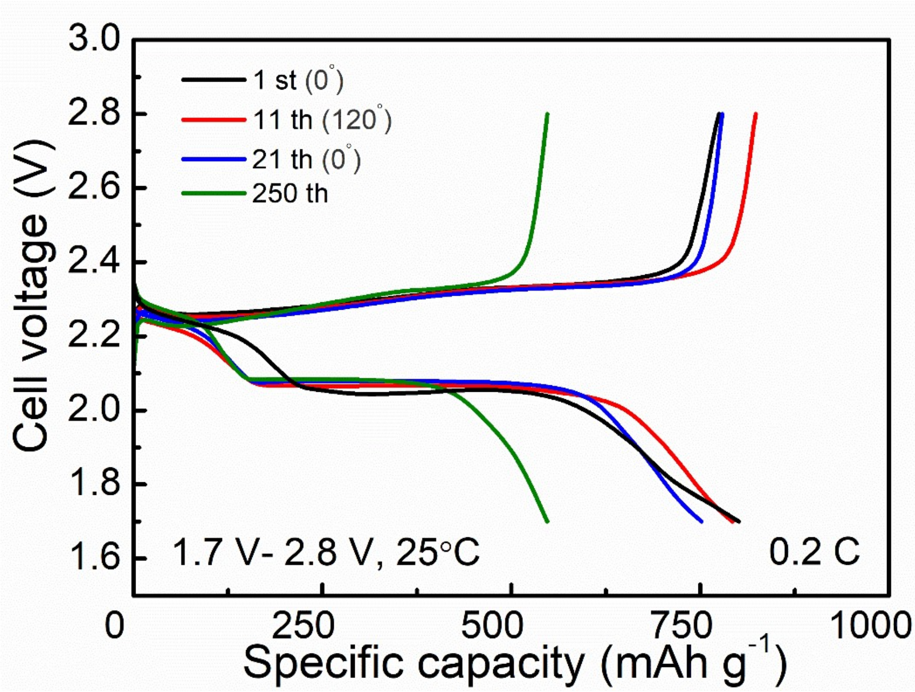

Fig. S7. Discharge-charge profiles of Co-TCN assembled Li-S pouch cell at 0.2 C.

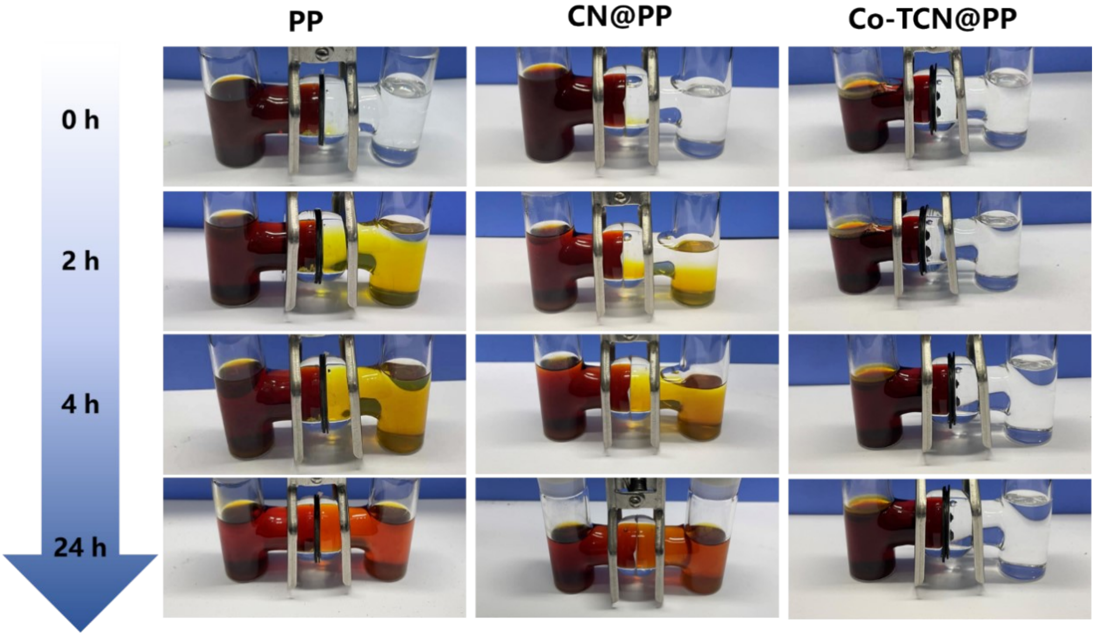

Fig.S8. Photographs of the glass cells with Li_2S_6 in DOL/DME solution and pure DOL/DME solvent in the left and right chambers, respectively, separated by pp, CN@PP or Co-TCN@PP.

Table S1. The setup parameter of coin cells and pouch cells.

**Coin cell test information**		
**Active material**	Cathode: Sulful (1675 mAh/g)	
**Additive**	Ketjenblack	
**Binder**	PVDF	
**Electrolyte**	1M LiTFSi and 1wt% $LiNO_3$ in DOL and DME (v:v,1:1)	
**Current collector**	Al	
**Separator**	Co-TCN@PP	
**Pouch cell test information**		
	Anode: Li	
	Cathode: S/KB	
**Comments**	We tested both pouch cells and coin cells. The S loading was controlled to 1 mg/cm^2.The highest can reach to 5.6 mg/cm^2. The area of the electrode was 1.3 cm^2 for coin cell and 4 cm^2 for pouch cell.	
**Measurements and Calculations**		
**1C = 1675 mA/g Capacity = current density*time (h)**		
**Initial capacity (mAh/g)**	863	
**Capacity achieved (mAh/g)**	621	
**@rate**	2C	
**Number of cycles tested**	400	
**Capacity retention**	71%	
**Testing temperature**	30℃	
